# Bioreduction: the biological activity, characterization, and synthesis of silver

**DOI:** 10.3906/kim-1910-8

**Published:** 2020-04-01

**Authors:** Nesrin KORKMAZ

**Affiliations:** 1 Department of Biotechnology, Faculty of Science, Bartın University, Bartın Turkey

**Keywords:** Silver nanoparticles, *Anthurium andraeanum*, antibacterial activity, antibiofilm activity

## Abstract

Today, nanoparticles are effectively used in different areas. Initially, physical and chemical methods were used in the synthesis of nanoparticles. Biosynthesis (green synthesis) has emerged as an alternative to overcome the toxic effects of chemically synthesized nanoparticles. In this study, green synthesis of silver nanoparticles (AgNPs) with the leaf extract of *Anthurium andraeanum* was performed. UV-Vis spectrophotometry, scanning transmission electron microscopy, and XRD were applied to characterize the biosynthesized nanoparticles. As a result of the characterization, the spectra showed that a spectrum at a wavelength of about 419 nm and a spherical size of 38 nm nanoparticles was formed. Antibacterial and biofilm inhibition activities of AgNPs against gram-positive and gram-negative bacteria were determined. AgNPs at a concentration of 1 mg/mL showed antibacterial activity against all of the bacterial strains. In the antibiofilm activity study, the highest inhibition percentage was obtained against the *P. fluorescens* strain at 87.1%, at a concentration of 0.5 mg/mL.

## 1. Introduction

Nanotechnology and nanoparticles are increasingly recognized for their applications in aerospace engineering, nanoelectronics, environmental improvement, medical health, and consumer products [1,2]. Nanoparticles, by definition, are structures with dimensions between 1 and 100 nm [3].

Unique nanometallic particles can significantly alter physical, chemical, and biological properties due to their surface/volume ratios. Therefore, these nanoparticles have been used for various purposes [4]. Various methods have been adopted for the synthesis of nanoparticles. The most common method for the synthesis of silver nanoparticles (AgNPs) is the chemical reduction by inorganic agents, the use of chemically synthesized AgNPs. The use of biogenic nanoparticles is gaining popularity to reduce potentially toxic effects [5]. In general, traditional physical and chemical methods appear to be very expensive and dangerous. Biologically prepared AgNPs show high yield, solubility, and stability [6]. Biological agents such as plant extracts, bacteria, fungi and yeasts without chemical toxicity can be used for the synthesis of AgNPs. This is a safe choice for both applications [7]. In recent years, such studies have been frequently encountered and are rapidly evolving to become an approach linking nanotechnology and biotechnology.

With the rapid development of nanotechnology, nanoparticle applications have been expanded. Nanomaterials are most commonly used in Ag-containing products [8–9]. In the field of medical applications, wound dressings, contraceptive devices, surgical instruments, bandages, and bone prostheses are covered or buried with nanosilver. Other uses of AgNPs include breathing apparatuses, household water filters, antibacterial sprays, cosmetics, detergents, and textile products [10–12].

AgNPs have long been known to have antibacterial properties. AgNPs exhibit more effective antimicrobial properties due to their large surface areas, which provide better contact with microorganisms. AgNPs adhere to the cell membrane and penetrate the bacteria. Bacterial membranes contain sulfur-containing proteins, and nanoparticles interact with phosphorous-containing compounds, such as those proteins and DNA in the cell. When the AgNPs penetrate into bacteria, they form a low molecular weight region in bacteria cell. These regions give rise to a complex DNA structure that causes abnormal conditions for the bacterial respiratory chain and cell division. These particles secrete silver ions that increase their bactericidal activity in bacterial cells [13].

*Anthurium andraeanum* is a flowering plant species. It belongs to the family Araceae. It is also used as an indoor ornamental plant. In this study, it was aimed to emphasize that ornamental plants can be used as an alternative for the synthesis of AgNPs and that there are endless bioreducers for biosynthesis reactions. Shazhni et al. conducted a study on the investigation of the phytochemical components of different parts of *A. andraeanum*. The plant, flower, leaf, stem, and root materials were extracted using different solvents, such as such as water, acetone, dimethyl sulfoxide, chloroform, and ethanol via phytochemical screening. As a result of the screening, they reported the presence of phenolic compounds and tannin compounds in aqueous extracts of green leaves, where alkaloids, flavonoids, and steroids were present in the flower aqueous extract of the plant. In the current study, using only the green leaves of the plant, phenolic compounds and tannin compounds were used as reductants in the synthesis of AgNPs, and these substances are known as antimicrobial agents against most bacteria [14].

This study was conducted on a more environmentally friendly and rapid synthesis of AgNPs. In this study, AgNPs were biologically synthesized using the leaf extract of *A. andraeanum* . UV-Vis, X-ray diffractometry, and SEM analyses were performed. The antibacterial activity of AgNPs on 8 bacteria, as well as the effect of antibiotics, was investigated.

## 2. Materials and methods

### 2.1. Plant extraction

*A. andraeanum* was used for the synthesis of biological nanoparticles. *A. andraeanum* leaves were collected and washed twice with distilled water to remove any possible contamination and then dried. The dried leaves were thoroughly ground in a porcelain mortar. Next, 30 g of the ground dried leaves were added into deionized water (100 mL) and mixed in a magnetic stirrer for 30 min. The mixture was then heated in a water bath at 60 °C for 10 min. After cooling to room temperature, it was centrifuged at 3500 rpm for 10 min and filtered through filter paper to obtain the plant extract.

### 2.2. Nanoparticle synthesis

The synthesis of AgNPs was performed with few changes in the working procedures of Rather [15] and Saini [16]. Briefly, the filtered *A. andraeanum* plant extract was used for the synthesis of AgNPs by adding 5 mL (1 mg/mL) to 95 mL of 1 mM of AgNo_3_ in an aqueous solution at 25 °C. The beaker was held in the magnetic stirrer for 5 h at 37 °C in the dark. A significant color change was observed within 24 h. The anoparticles were separated from the mixture using centrifugation (for 10 min at 3500 rpm). The supernatant was discarded and the precipitate was resuspended in deionized water and centrifuged again to avoid uncoordinated biomolecules. The characterization stage of the AgNPs was observed by the expected absorbance pick of Ag°, the mean of the AgNP diameter was calculated using the XRD data. Morphology and particle size were determined by SEM and scanning transmission electron microscopy (STEM).

### 2.3. Biological activity

Biosynthesized AgNPs were applied to determine their antibacterial activity against *Enterobacter aerogenes* , *Salmonella infantis* , *Salmonella typhimurium* , *Escherichia coli* CFAI, *Enterococcus faecalis* , *Staphylococcus aureus* , *Staphylococcus epidermidis* , and *Bacillus subtilis* . The minimum inhibitory concentration (MIC) assay was used to calculate the lowest concentration that inhibited bacterial growth. Another test called the minimum bacteriostatic/bactericidal concentration (MBC) was used to determine the bacterial inhibition in the wells where no observable bacterial growth was observed in the MIC assay. Bacterial strains were inoculated in Luria Bertani (LB) broth until reaching 0.5 McFarland standard turbidity (1.5 ×108 CFU/mL). The AgNP solution was prepared as a 1 mg/mL concentration. At the same volume, the LB broth and AgNPs solution were added into the first well of a 96-well microplate, and 2-fold dilutions were performed from 1 mg/mL to 0.0312 mg/mL. Next, the dilutions were incubated at 37 °C for 24 h. The growth of bacteria was measured by UV-Vis spectrophotometry at 600 nm (OD600). After analyzing the values, samples for the minimum bactericidal/bacteriostatic concentration (MBC) were selected and they were streaked onto LB-agar plates and incubated at 37 °C overnight. The following day, the growth patterns were checked and determined as either bactericidal or bacteriostatic [17].

The biofilm inhibition effect of the AgNPs was analyzed against 3 gram-positive ( *Bacillus subtilis* , *Staphylococcus aureus* , *Enterococcus faecalis* ) and 3 gram-negative bacteria ( *Escherichia coli* CFAI, *Pseudomonas fluorescens* , *Salmonella enteritidis* ). Bacterial growth was standardized to 0.5 McFarland standard turbidity (1.5 ×108 CFU/mL). The AgNPs were initially tested at 1 mg/mL in 96-well microplates and serially diluted to 0.0312 mg/mL. All of the mixtures were incubated at 37 °C for 48 h and then drained. All of the wells were washed with d-H2 O and left to dry at room temperature. Next, 95% methanol was added for 15 min and then removed. As the next step, 0.1% crystal violet was added into the wells and incubated for 10 min. All of the wells were elutriated until the stain was completely removed. Next, 33% glacial acetic acid was added onto the gram-positive bacteria and 95% ethyl alcohol was added onto the gram-negative bacteria, and they were then incubated for 15 min. Afterwards, the growth of bacteria was measured by UV-Vis spectrophotometry at 600 nm (OD600) and then the percentage of biofilm formation was calculated using the following formula:

%Inhibition = (Control OD600 – Sample OD600) / Control OD600 ×100 [18].

## 3. Results and discussion

### 3.1. UV-Vis absorption studies

**Figure 1:**
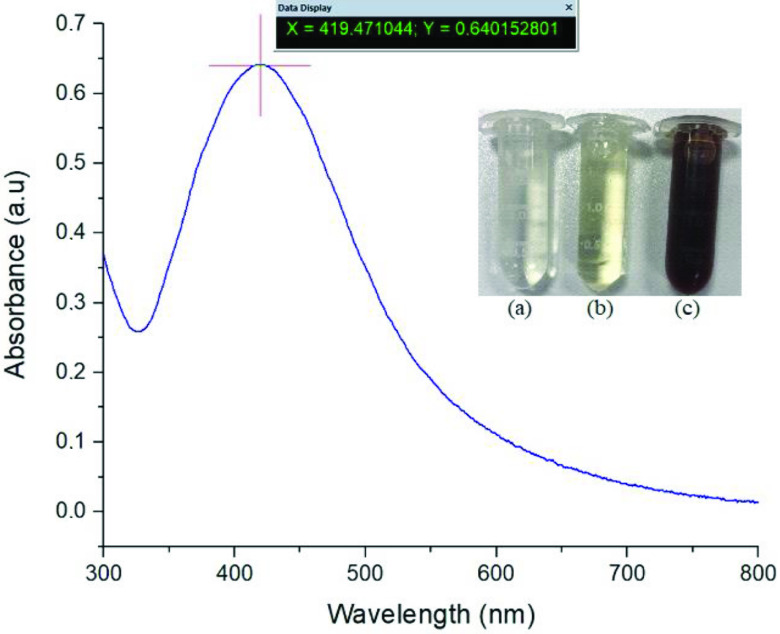
UV-Vis absorption spectra of the AgNPs: (a) AgNo_3_ solution, (b) plant extract, and (c) AgNP solution.

When *A. andraeanum* leaf extract was added to AgNo_3_ aqueous solution, the Ag^+^ ions were reduced to AgNPs. The reaction showing the formation of AgNPs in the mixture after approximately 40 min was observed by conversion of the color of the solution from yellow to dark brown. In the literature, it was reported that the color of the solution changes from yellowish to hues of brown due to the stimulation of the surface plasmon vibration in metal nanoparticles [19]. The UV-Vis absorption spectrum of the AgNPs in the presence of *A. andraeanum* leaf extract is shown in Figure 1, where it can be seen that the absorption spectrum of the AgNPs that formed in the reaction medium had an absorbance peak at 419 nm.

### 3.2. XRD analysis

The high absorbance optical density value showed that the conversion of Ag^+^ to Ag° as a nanoparticle was high. In the biosynthesis of metal nanoparticles, this is a bottom-up approach because of the occurrence of nuclear reactions, such as reduction-oxidation. In general, there are 2 possible ways of converting AgNo_3_ into AgNPs using biological methods. First, the plant may have an effective conversion from AgNo_3_ to AgNPs due to the organic materials extracted [20]. The present results reinforced the idea that organic substances contained in the plant extract were responsible for the synthesis of AgNPs.

**Figure 2:**
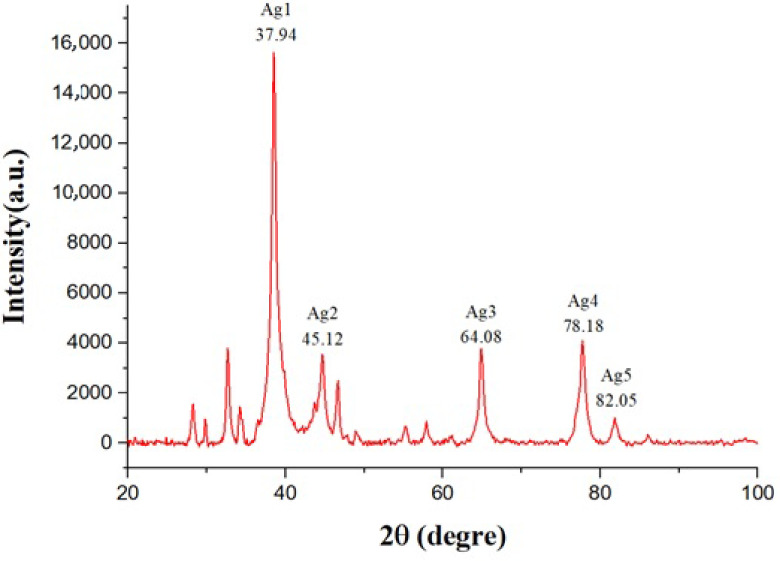
X-ray diffraction pattern of the AgNPs.

UV-Vis spectral analysis was performed to verify the results and understand if the crystalline structure of the particles was reduced to Ag°; a sample of *A. andraeanum* plant extracts exposed to Ag^+^ ions was examined using XRD. The XRD data of the AgNPs obtained from biosynthesis reduction of AgNo_3_ is shown in Figure 2. Planar spacing for the values of 2.32612, 2.02761, 1.42699, 1.21911, and 1.18135 Å (d computed) values of 37.94°, 45.12°, 64.08°, 78.18°, and 82.05°to 5 main peak correspond to planes. Other observed peaks were thought to belong to some organic residues present in the *A. andraeanum* leaf extract. The average particle diameter of the AgNPs was calculated using the following the Scherrer equation:

(1)D=(K λ)/(β cosθ)

This equation utilizes the reference peak width at angle θ , where λ is the X-ray wavelength, β is the width of the XRD peak at half height, and K is a shape factor constant = 0.9. By calculating the XRD data results, the particle size was approximately 38 nm. These results were consistent with standard silver values [21].

### 3.3. SEM and STEM study of the AgNPs

**Figure 3:**
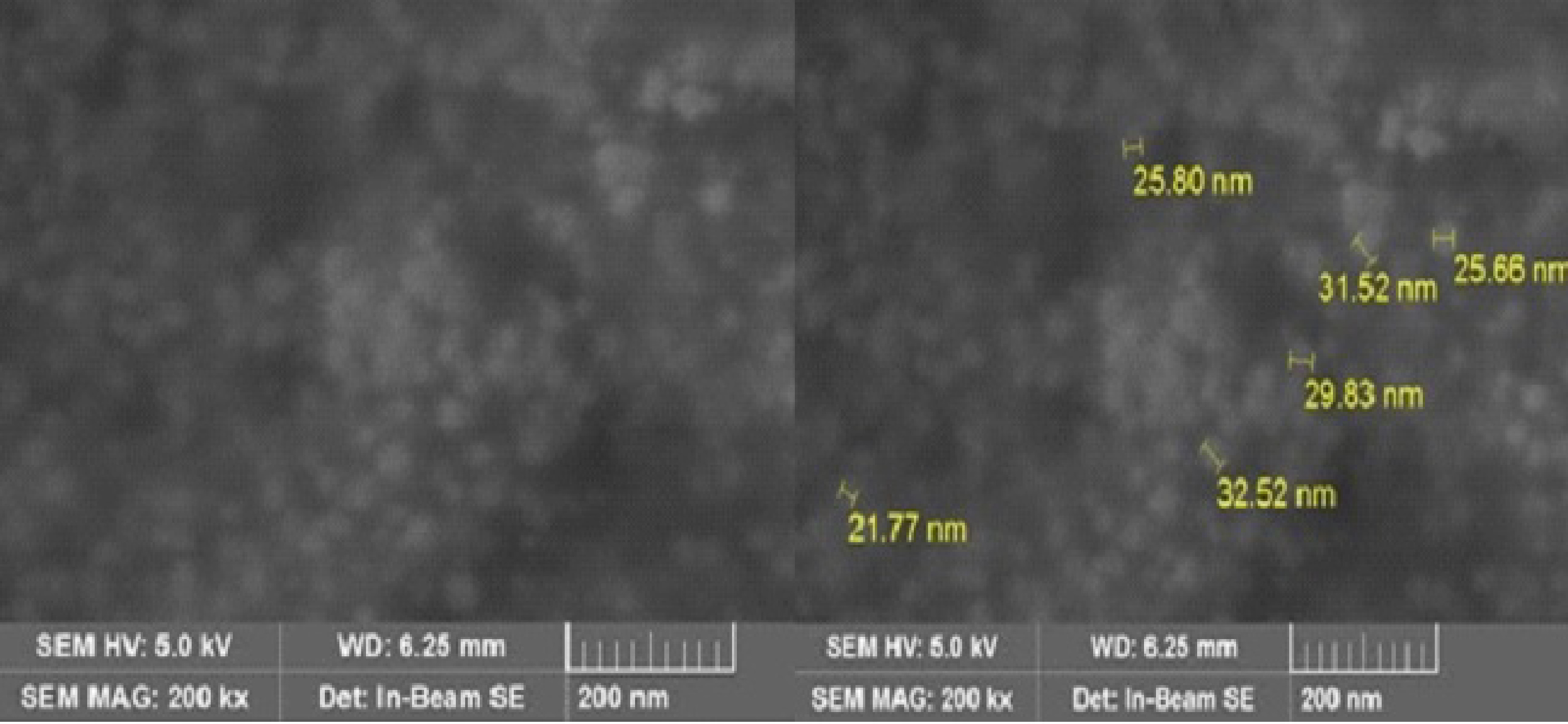
SEM of the AgNPs.

SEM images of the synthesized AgNPs were taken to determine the particle size and shape of the nanoparticles. SEM images of the AgNPs at 200 k magnification are shown in Figure 3.

The AgNPs had different sizes and shapes, ranging from 21 nm to 32 nm (Figure 3). Similarly, the particle size of the AgNPs synthesized using *Pelargonium graveolens* leaf extract was reported as 16–40 nm [22].

**Figure 4:**
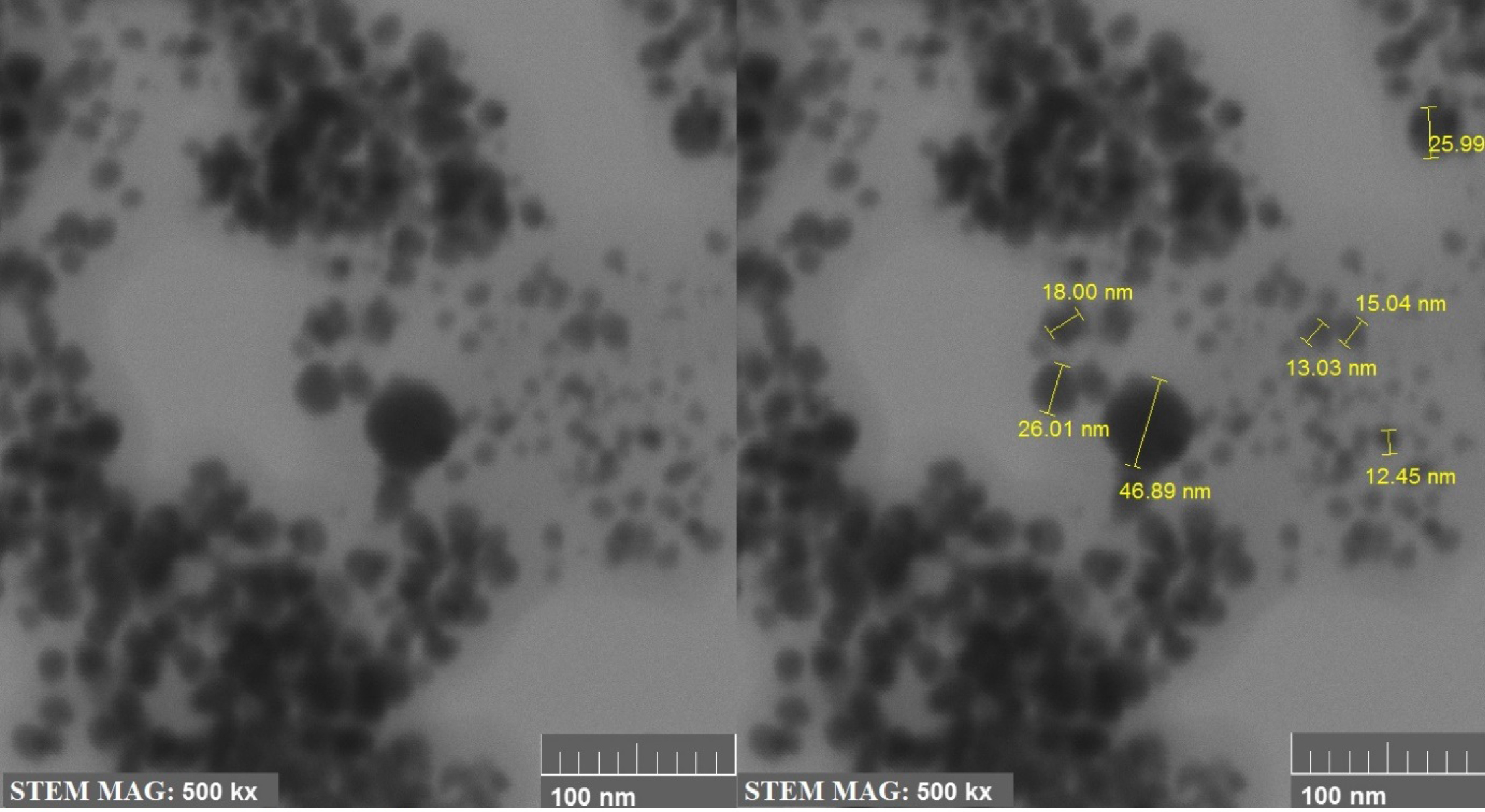
Size and morphology of the AgNPs analysis by STEM.

In order to see the shape and size of the AgNPs obtained using *A. andraeanum* plant extract, STEM analysis was also performed in addition to the SEM analysis (Figure 4). STEM analysis provided better results with 500 kx magnification at 100 nm. As can be seen in Figure 4, the nanoparticles provided integrity via their spherical shape. Their sizes ranged from 12 nm to 46 nm. It can be said that this had consistence with the average size obtained via X-Ray analysis.

### 3.4. Biological activity

Silver is a naturally antimicrobial material and due to its characteristics, and it has been used widely in medicine, food storage, cosmetic products, dye reduction, the textile industry, and environmental applications [23–25]. Using *A. andraeanum* extract, AgNPs were synthesized, and the MIC values and MBCs of the AgNPs were determined by testing the bacteria formation with various dilutions of AgNPs, ranging from 1 mg/mL to 0.0312 mg/mL. At different concentrations, they were found to possess antimicrobial activity against 8 gram-negative and -positive bacteria strains (Table 1), and the MBC was used to determine their activity as bactericidal or bacteriostatic. The AgNPs indicated a significant effect against all of the tested gram-negative bacteria at 0.125 mg/mL (MIC value). The MBC value was obtained against *S. infantis* and *E. coli* at 1 mg/mL, and *E. aerogenes* at 0.25 mg/mL, respectively. For the gram-positive bacteria strains, the AgNPs indicated inhibition activity against *S. aureus* and *B. subtilis* at 0.0625 mg/mL, and *E. feacalis* and *S. epidermidis* at 0.125 mg/mL. With the exception of *S. aureus* , the AgNPs showed a bactericidal effect on all of the gram-positive bacteria at 1 mg/mL.

**Table 1:** MIC and MBC concentrations of the AgNPs.

	1 mg/mL	0.5 mg/mL	0.25 mg/mL	0.125 mg/mL	0.0625 mg/mL	0.0312 mg/mL
*E. aerogenes*				M		
*S. infantis*	*			M		
*S. typhimurium*				M		
*E. coli* CFAI	*			M		
*E. faecalis*	*			M		
*S. aureus*		*			M	
*S. epidermidis*	*			M		
*B. subtilis*	*				M	

M: MIC, *: MBC.

Jayaprakash et al. (2014) reported the antibacterial effect of AgNPs from *Piper nigrum* extract using the disk-diffusion method. When compared to their antibacterial results against *E. coli* , *S. aureus* , and *B. subtilis* , the results of current study were similar [26]. Dipankar and Murugan (2012) stated that AgNPs synthesized from Iresine herbstii leaves demonstrated antimicrobial activity against *S. aureus* , *P. aeruginosa* , *E. coli* , *E. faecalis* , and *K. pneumonia* bacteria, which was compatible to the AgNPs prepared in the current study [24]. Malabadi et al. (2012) performed AgNPs from *Clitoria ternatea* plant extraction on gram-positive and -negative bacteria. They reported an important antibacterial effect. A thin layer of peptidoglycan in gramnegative bacteria would enhance the NPs invading cell. Ag^+^ ions strongly interact with enzyme thiol groups and disable them. Moreover, the structure of ROS causes cell membrane disruption, protein denaturation, and DNA losses. Consequently, these reasons indicated that AgNPs either inhibit bacterial growth or kill bacteria [27].

Biofilm is a layer of microorganisms that are an aggregation of bacterial substances, such as polysaccharides, nucleic acids, carbohydrates, and proteins. Some difficulties induced by the biofilm layer have presented a threat to the environment and human health in many areas, such as water supply networks, wastewater treatment plants, the food industry, and paper production [28]. According to the results of the current study, the AgNPs destroyed the biofilm layer of all of the tested bacteria. The highest antibiofilm activity percentage 87.1%, at a concentration of 50 mM, was shown against *P. fluorescens* (Table 2). Kalishwaralal et al. (2010) reported that the inhibition of *E. coli* biofilm formation was obtained with a concentration of 10 mM at the highest percentage [29]. Velazquez et al. (2015) reported that AgNPs inhibited the bacterial biofilm layer by impregnated dressing with AgNPs using the same bacteria [30]. In the current study, the biofilm formation of these 2 bacteria was vigorously inhibited. Gurunathan et al. (2014) declared that the antibiofilm activity of AgNPs against *P. aeruginosa* , *S. pneumonia* , *S. flexneri* , and *S. aureus* , gave rise to inhibition percentages similar to the results obtained in the present study [6].

**Table 2:** Biofilm inhibition percentage of the AgNPs.

	1 mg/mL	0.5 mg/mL	0.25 mg/mL	0.125 mg/mL	0.0625 mg/mL	0.0312 mg/mL
*P. fluorescens*	81.4	87.1	85.5	0	0	18.15
*E. faecalis*	43.95	53.75	42.6	0	0	0
*S. enteritidis*	38.15	47.55	43.5	28.85	0	1.25
*S. aureus*	14.75	40	35.6	0	0	6.8
*B. subtilis*	26.60	51.85	30.50	28.40	13.25	1.89
*E. coli* CFAI	24.56	30	41.5	23.15	0.0	0

Biosynthesized nanoparticles demonstrated antibacterial and antibiofilm activity against the tested bacteria in the current study. Ionic silver strongly interacts with thiol group of enzymes and inactivates them [31,32]. Specifically, inactivated enzymes might cause inhibition of DNA replication and bacterial growth. Moreover, the size and shape of the AgNPs might be problem for bacterial growth. They might also interact with cell walls and organelles, and either inhibit or kill bacteria strains [33].

In conclusion, it was determined that the nanoparticles synthesized from the plant extract showed antibacterial activity against some gram-positive and -negative bacteria. Moreover, syntheses of the AgNPs were eco-friendly; thus, it can be a choice instead of chemical antibacterial agents.

## 4. Conclusion

This study was performed to find natural, environmentally friendly, and easily available plant-based agents for the synthesis of metal nanoparticles. Phytochemicals found in *A. andraeanum* leaf extract are believed to reduce silver ions in metallic nanoparticles.

AgNPs can be used in wound ointment, silver-plated textiles, and especially for burn injury treatment, due to their antimicrobial effect. Nanoparticles obtained via biosynthesis can also be used to prevent biofilm formation in the food industry and water treatment plants. To clarify the antibacterial mechanisms of Ag-NPs, more detailed analyses are required. In general, these AgNPs can be an alternative for the control of antibiotic-resistant microorganisms. The data presented in the current study contributes to new and unexplored nanomaterial for literature.
